# Impact of COVID-19 pandemic on prescription of psychotropic medications in the Italian paediatric population during 2020

**DOI:** 10.1186/s13052-024-01670-5

**Published:** 2024-05-20

**Authors:** Francesca Gallinella, Francesco Trotta, Filomena Fortinguerra

**Affiliations:** Italian Medicines Agency (AIFA), Via del Tritone, 181, Rome, 00187 Italy

**Keywords:** Adolescent, Child, Outpatients, Drug utilization, Psychotropic drugs

## Abstract

**Background:**

There is a global perception that psychotropic utilization in children and adolescents is increasing, especially with the onset of COVID-19 pandemic. Available literature data on paediatric psychotropic medication prescriptions in Italy are limited to one or few regions and not updated. The aim of this study was to provide updated data on psychotropic prescriptions referred to the whole Italian paediatric population, as overall and by subgroups of medications and to evaluate if the COVID-19 pandemic during 2020 had an impact on prescription rates.

**Methods:**

A descriptive study on psychotropic drug utilization in children and adolescents (< 18 years) resident in all Italian regions during 2020 was performed. Patients registered in the Pharmaceutical Prescriptions database with at least one prescription/dispensing of a psychotropic medication (antipsychotics-N05A), (antidepressants-N06A) and (psychostimulants-N06BA) during the study period were considered. The indicators used were the prescription rate (number of prescriptions per 1000 children) and prevalence of use (proportion of the paediatric population with at least one prescription in the relevant year).

**Results:**

During the 2020 the prevalence of psychotropic drug use in the paediatric population was 0.3%, increased of 7.8% if compared to 2019. The same trend was observed for the prescription rate, which recorded an average of 28.2 per 1000 children with an increase of 11.6% if compared to previous year, representing the 0.6% of the overall drug use in this age group. The data showed a growing trend prescription by age, reaching the peak in adolescents aged 12–17 years old, with a prescription rate of 65 per 1000 children and a prevalence of 0.71%. Considering the subgroups of psychotropic medications, the highest prevalence of use was found for antipsychotic drugs, received by the 0.19% of the paediatric population during 2020.

**Conclusions:**

Psychotropic drug utilization in children and adolescents has grown during 2020 in Italy and worldwide, raising alarms from health care clinicians and patient advocates about the increase of burden of mental diseases in paediatric population during the COVID-19 pandemic. A more systematic monitoring of the use of psychotropic medications should be implemented in all countries for collecting relevant information about children and adolescents taking psychotropic drugs, in order to address the present and the future of the mental health of the paediatric population.

## Introduction

Most adult mental health disorders begin in childhood or adolescence with a severe and chronic course that can seriously disrupt sensitive developmental periods with lifelong adverse consequences in adult age. An increase in the prevalence of mental health disorders in children and adolescents has already been observed over the past 30 years, with different estimates ranging from 10 to 18%, depending on the geographical setting, the age range, and the tools used for the diagnosis [1]. Data referring to 2019 estimated that the 8.8% of children and adolescents has been diagnosed with a psychiatric disorder worldwide, accounting for 13% of the global burden of disease in this age group [2, 3]. In Italy the Italian preadolescent mental health population project (PrISMA-Progetto Italiano Salute Mentale Adolescenti) performed in 2006 estimated that the prevalence of cases detected by the child behavior checklist (CBCL) in the population 10–14 years of age was 9.8%, while, using DSM-IV criteria, the estimate was 8.2% [4].

Several studies suggested a negative psychological impact of the COVID-19 pandemic quarantines on mental health of the paediatric population worldwide, with an increasing number of the reported new diagnosis of mental disorders in children and adolescents over time since the outbreak in March 2020 [5–11]. Anxiety, depression, sleep disorders, and eating disorders resulted to be among the leading causes of illness and disability in this population [12–21]. Moreover, children with pre-existing behavioral disorders, such as autism spectrum disorders or ADHD had a high probability of worsening of their behavioral symptoms, probably also due to difficulties in accessing to child neuropsychiatry support services for pandemic containment measures [22–31]. This situation was confirmed by an increased number of visits in child and adolescent neuropsychiatric emergency departments after the first weeks of COVID-19-induced social lockdown [32–34].

Several studies on drug utilization performed in pre-pandemic periods had already reported an increase of prescription rates of psychotropics in children and adolescents worldwide, with the consumption in the United States of America (USA), being markedly higher than that reported in European (EU) countries [35–38]. The COVID-19 outbreak accentuated these increasing trends globally, as it was documented by several international studies currently available [39–51].

In Italy some studies based on surveys or interviews involving small samples of children and adolescents and their parents suggested that the prolonged quarantine period during COVID-19 pandemic significantly affected their emotional wellness causing severe emotional and behavioral difficulties, sleep habits, anxiety and mood symptoms [52–55]. Since the negative effects of stress experienced during school closures, social distancing and isolation on mental health and well-being of children and adolescents may manifest later on during development, it would be crucial to monitor the related potential long-term adverse outcomes [56]. Underestimating the impact of COVID-19 on mental health of young people runs the risk to become a healthcare emergency, considering the already troublesome situation in Italy and elsewhere in terms of capacity of neuropsychiatric services to manage childhood and adolescent neurodevelopmental disorders [48, 57].

Available literature on psychotropic medication prescriptions in children and adolescents in Italy are currently limited to one or few regions and not updated [58–60]. The last study representative of the Italian paediatric population was published in 2016 reporting data from seven Italian regions until 2011 [61]. In addition, no data were currently available on drug utilization during and after pandemic in Italy. This study aimed to provide updated data on psychotropic medication prescriptions referred to the whole Italian paediatric population, as overall and by subgroups of medications (antipsychotics, antidepressants, and psychostimulants) during 2020 to evaluate if the COVID-19 pandemic had an impact on prescription rates of these medications in Italy.

## Methods

A descriptive study on psychotropic drug utilization in children and adolescents (< 18 years) in Italy resident in all Italian regions during 2020 was performed. Patients registered in the Pharmaceutical Prescriptions database (also known as the Italian Health Insurance Card database) with at least one prescription/dispensing of a psychotropic medication during the study period were considered. The Pharmaceutical Prescriptions database is an administrative healthcare database, which collects patient-level data on all medicines reimbursed by the National Healthcare Service (NHS) and dispensed by community pharmacies to whole Italian population (patients resident in all Italian regions) in order to evaluate the use of medications in the Italian population by age, gender, and region of residence. Only psychotropic medications reimbursed by the Italian NHS and dispensed by community pharmacies in the outpatient setting were considered in the analysis. Data on drug dispensation were arranged according to the Anatomical Therapeutic Chemical (ATC) classification established by the World Health Organization Collaborating Centre (WHOCC) for Drug Statistics Methodology as follows: N05A (antipsychotics), N06A (antidepressants) and N06BA (psychostimulants). Other psychotropic drugs such as anxiolytics, hypnotics and sedatives are not reimbursed by the NHS, and thus were excluded from this study.

The indicators used to report psychotropic medication use in the paediatric population were the prescription rate (expressed as number of prescriptions per 1000 children) and prevalence of use (the proportion of the paediatric population with at least one prescription in the relevant year). For the calculation of percentage of consumption on overall paediatric consumption and between male and female the drug consumption was measured as the number of defined daily doses (DDDs), which is the assumed average maintenance dose per day for a drug used for its main indication in adults [62].

## Results

During 2020 the prevalence of psychotropic drug use in the paediatric population was 0.3%, increasing of 7.8% if compared to 2019 (Table 1). The same trend was observed for the prescription rate, which recorded an average of 28.2 per 1000 children with an increase of 11.6% if compared to previous year, representing the 0.6% of the overall drug use in this age group. The consumption (expressed as DDDs) was higher in males than females (59% vs 41%).

**Table 1:** Prescription of psychotropic medications in paediatric population during 2020

**Users (N.)**	32,80
Prevalence (%)	0.3
Δ % 20 − 19	7.8
**Prescriptions (N.)**	269,03
Prescriptions per 1000 children (N.)	28.2
Δ % 20 − 19	11.6
% on overall drug consumption (measured as DDDs)*	0.6
**Packs (N.)**	297,75
Packs by prescription (N.)	1.1
Packs per1000 children (N.)	31.2
% F/M (measured as DDDs)*	41/59

* calculated on overall consumption of psychotropic medications in paediatric population

**Fig. 1:**
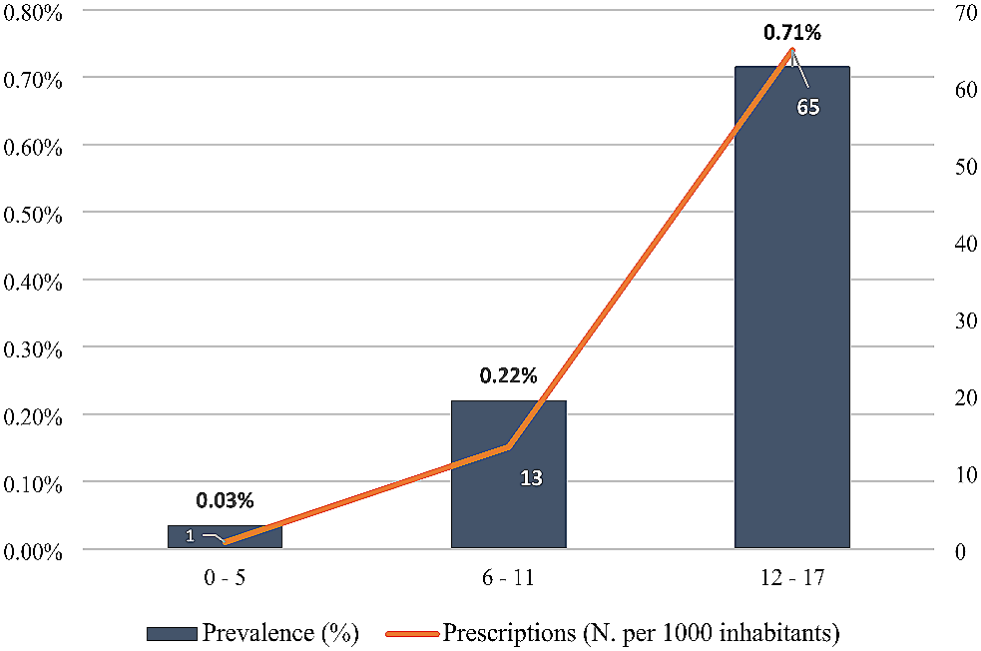
Prevalence (%) and prescriptions rate (N. per 1000 inhabitants) of psychotropic medications in paediatric population by age class during 2020

**Fig. 2:**
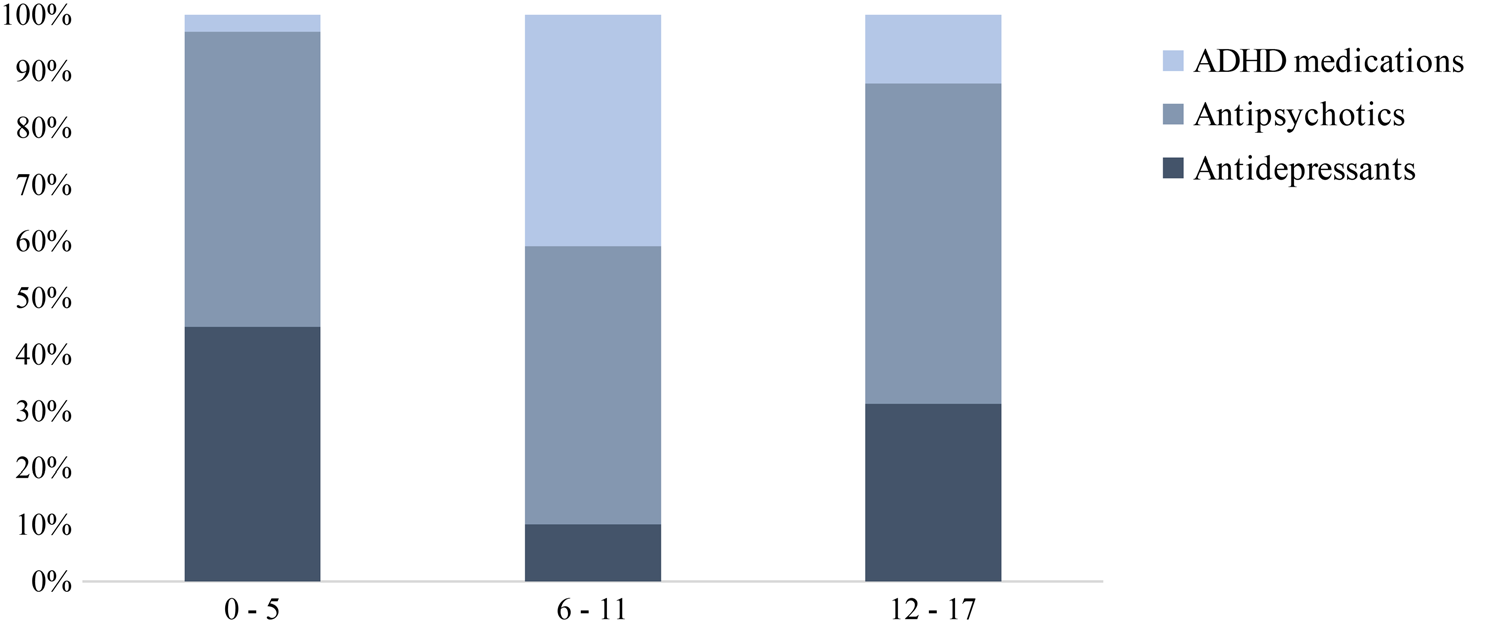
Distribution (%) of psychotropic medication prescriptions in paediatric population by age class during 2020

The data showed a growing trend prescription by age, reaching the peak in adolescents aged 12–17 years old, with a prescription rate of 65 per 1000 children and a prevalence of 0.71% (Fig. 1). Considering the subgroups of psychotropic medications, the highest prevalence of use was found for antipsychotic drugs, received by the 0.19% of the paediatric population during 2020 (Table 2). The number of prescribed packs for anti-psychotics, equal to 15.7 per 1000 children, has recorded an increase of 17.2% compared to the previous year. Antidepressants were found at second place, with a prevalence of 0.14% and the prescription of 8.1 packs per 1000 children, which were increased of 6.4% and, lastly, ADHD medications, represented by methylphenidate and atomoxetine, which had a prevalence of 0.06% and a prescription rate of 4.4 packs per 1000 children, which was increased of 3.1%. Considering that most of the psychotropic drug prescription are concentrated in the age group 12–17 years, the percentage distribution of consumption among the different subcategories shows that the prescription of antipsychotics is high in all age groups, while ADHD medication were concentrated in the age group 6–11 years (Fig. 2).

**Table 2:** Prevalence (%) and consumption (N. packs per 1000 inhabitants) of psychotropic medications in paediatric population by drug class during 2020

Drug class	Prevalence (%)	Packs per 1000 children	
		*N*.	Δ% 20 − 19
Antipsychotics	0.19	15.7	17.2
Antidepressants	0.14	8.1	6.4
ADHD medications	0.06	4.4	3.1
**Overall**	**0.30**	**31.2**	**11.6**

Among the antipsychotic drugs, risperidone was the one with the highest prevalence of use: 0.09%, with 4.14 prescriptions per 1,000 children (Table 3). It is licensed for the short-term (up to 6 weeks) treatment of persistent aggression in conduct disorder in children from the age of 5 and adolescents with intellectual disabilities or with sub-average intellectual functioning, in which the severity of aggressive behaviours or other disruptive behaviours requires a pharmacological treatment. Risperidone is also used in children and adolescents with autism spectrum disorder. The second most widely used drug - with 0.07% of prevalence of use and 6.08 prescriptions per 1000 children - was aripiprazole, an antipsychotic authorized to treat schizophrenia from 15 years of age and moderate to severe manic episodes of bipolar disorder in adolescents from 13 years of age. Risperidone and aripiprazole, among the atypical antipsychotics, are the only drugs approved by US Food and Drug Administration (FDA) for the treatment of severe behavioural problems associated with autism spectrum disorder.

**Table 3:** Ranking of the first ten psychotropic medications by prevalence in paediatric population during 2020

Drug	Prevalence (%)	Packs per 1000 children		% DDD*	
		*N*.	∆ % 20 − 19	Male	Female
risperidone	0.09	4.14	3.9	78.9	21.1
aripiprazole	0.07	6.08	15.5	63.1	36.9
sertraline	0.06	4.26	6.2	44.0	56.0
methyilphenidate	0.06	4.96	1.8	88.4	11.6
fluoxetine	0.03	1.69	11.4	30.6	69.4
quetiapine	0.02	1.14	6.1	40.7	59.3
olanzapine	0.01	1.07	13.1	55.6	44.4
lithium	0.01	1.42	22.0	48.4	51.6
paroxetine	0.01	0.37	-6.3	37.4	62.6
escitalopram	0.01	0.40	-2.3	33.6	66.4

* calculated on overall DDD of psychotropics in paediatric population

The third most prescribed medication, with 4.26 prescriptions per 1000 children, was sertraline, a serotonin reuptake inhibitor (SSRI) antidepressant licensed for the treatment of obsessive-compulsive disorder (OCD) in children and adolescents aged 6–17 years old, while the fourth most prescribed drugs was represented by methylphenidate, a psychostimulant included in a multimodal treatment program for ADHD in children from 6 years of age and adolescents, with the number of prescriptions equal to 4.96 per 1000 children during 2020. Consumption of risperidone, aripiprazole and methylphenidate is higher in males than in females, while for sertraline most users are females.

## Discussion

In Italy the prevalence of psychotropic drugs in paediatric population during 2020 was about 3‰ at national level, increased by 7.8% if compared to the previous year. This value was also higher than that resulting from the last multiregional study conducted in Italy on a representative sample of half of the Italian paediatric population with data referred to 2011 (about 2‰) [61]. Among the different classes of psychotropic drugs analyzed, the antipsychotics registered the highest increase in the prescribing, while for the antidepressants and ADHD medications the increases were smaller.

The greatest evidence supporting the use in paediatric population is for the pharmacological treatment of ADHD, a psychiatric disorder for which treatment with methylphenidate is still the reference drug to date [62–65]. Indeed, pharmacological treatment is used only in cases in which the psycho-social interventions adopted or the psycho-behavioral therapies undertaken have proved insufficient. Moreover, methylphenidate must be administered by a child and adolescent neuropsychiatrist, or related specialist in charge of the territorial centres of child neuropsychiatry, under the supervision of a regional referral centre for the diagnosis and treatment of ADHD, based on of a specific Therapeutic Plan, compliant with national and European guidelines. To date there is also reasonable evidence addressing the selective serotonin reuptake inhibitors (SSRIs) for the treatment of moderate to severe Major Depression Disorder (MDD), OCD and anxiety disorders, atypical antipsychotics for the treatment of early-onset schizophrenia (EOS) and related psychosis, and risperidone for autism [66].

Despite the increase observed in 2020 in Italy, the comparison with published international epidemiological studies performed at the onset of the COVID-19 pandemic showed that the prescription of psychotropic medications in Italian paediatric population resulted still much lower than those observed in the USA, Australia and in some EU countries [42, 45, 46, 49, 51].

In USA the proportion of youths with a psychotropic prescription increased after COVID-19 outbreak, peaking at 36.9% in April 2020, with the largest for ADHD medications (22.4%), followed by antidepressants (16.0%), antipsychotics (6.1%), and mood stabilizers (4.3%). When comparing April 2020 − 2019, antipsychotics had the largest year-over-year percent change (41.9%), followed by antidepressants (37.9%), mood stabilizers (34.4%), and ADHD medications (20.4%); in September 2020, when the number of treated youths was similar to pre-pandemic, the year-over-year percent change in ADHD medication prescription decreased (− 2.8%), while other psychotropic classes increased between 3.1% and 5.0% compared to September 2019 [45, 46]. A marked increase in psychotropic medications use in children and adolescents was also observed in Australia [47, 48], with a prevalence passing from 33.8 to 60.0 per 1,000 children between 2013 and 2021 [49].

Among the EU countries, Denmark, which already experienced an increase in prescriptions number from 1.3 to 12.4 per 1000 children between 1996 and 2010, showed an increase of 37% in rates of incident users of any psychotropic medication among adolescents aged 12 to 17 years with or without a psychiatric history within the last 5 years during the COVID-19 pandemic [50].

In France random samples from the French National Health Insurance (CNAM) of children under 17 showed that 2.5% had been prescribed a psychotropic drug prescription in 2010 [67]. A more recent study performed during COVID-19 pandemic period showed an increase in users of antidepressants (+ 0.80%), anxiolytics (+ 0.59%), and hypnotics and sedatives (+ 2.55%) across the people aged 12–18 since March 2020 compared to the period from January 2015 to February 2020 [51].

In Italy, the prevalence of psychotropic prescriptions for children and adolescents showed a decrease from 2006 to 2011 (from 1.15 to 1.03 per 1000 children) [38], but an increase to 3 per 1000 children during 2020 as we found in this study.

According to our study results, the emergence of the COVID-19 pandemic in 2020 may have created an environment in which many determinants of poor mental health outcomes have been exacerbated resulting in an increase of psychotropic medications prescriptions, especially antipsychotics, consistently with what is reported in the literature about the increase the global burden of mental disorders in both adult and paediatric population [68].

Evidence on short-/medium-term impacts of COVID-19 on mental health worldwide currently confirms that children and adolescents suffering from pre-existing mental disorders, as well as those who developed mental health symptomatology during or immediately after pandemic, are most exposed to significant changes in mental health symptomatology [69]. The risk for a substantial mental health burden highlights the importance of further research in this area. In this context, monitoring the prescriptions of psychotropic medications in pediatric population is crucial to identify timely the emerging or worsening of mental health difficulties and to minimize the potential long-term effects of the COVID-19 pandemic on mental health of young people, even in specific high-risk subgroups [19]. International studies involving comparison between countries would also be of interest to understand how different health policies with regard to the handling of the pandemic had influenced the mental health of children and adolescents [70].

### Strengths and limitations of the study

Our study presents some limitations typically characterizing studies relying on data collected in administrative drug prescription databases; firstly, the lack of information concerning the diseases for which medications were prescribed, which does not allow us to make any further considerations on their use; secondly, our data were referred only to prescription of medicines dispensed in community pharmacies and reimbursed by the Italian NHS, thus excluding non-reimbursed medications (i.e. benzodiazepines) and the access to non-pharmacological therapies, consequently we were not able to investigate the appropriate use of these medications. Furthermore, the analyzed data could not represent the true medication intake, since we assumed that the prescription dispensed was administered to the patients. In case the medicine is dispensed but not actually taken by patient, the medication use could be over-estimated. However, since several studies based on administrative data considered the dispensation as a good “proxy” measure for medication use [71], our study can provide an adequate estimate of drug utilization of psychotropics. In fact, to our knowledge, this is currently the largest study illustrating the prescription data on psychotropic medications in the Italian paediatric population and the first data related to the impact of COVID-19 pandemic on psychotropic drug utilization in this age group, since previous studies were limited to smaller samples [58–60] or few Italian regions [61]. On the other hand, the availability of individual-level drug prescription data covering the whole Italian paediatric population allowing us to investigate the drug prescriptions by age, sex and region could be considered a very strong point for this study. Nevertheless, we were not able to fully investigate the drug prescriptions patterns, in order to better characterize drug users and medical prescribers’ habits in order to evaluate their optimal use in clinical practice.

## Conclusions

Psychotropic drug utilization in children and adolescents has grown in Italy during 2020 consistently with what emerged by international studies worldwide, raising alarms about the increase of burden of mental diseases in paediatric population after the COVID-19 pandemic. Since a very heterogeneous reporting is common across drug utilization studies [72], a more systematic monitoring of the use of psychotropic medications in children and adolescents should be implemented in all countries for collecting information, which can really help to address the present and the future of the mental health of the paediatric population.

## Data Availability

All data generated or analysed during this study are included in this published article.

## Declarations

### Authors’ information

The views expressed by authors in this article are their personal views and may not be understood or quoted as being made on behalf of or reflecting the position of the authors’ organization.
